# Multiomics Biomarkers of ZnO Nanoparticle Toxicity Using Mitochondrial, Oxidative Stress and Epigenetic Signatures

**DOI:** 10.3390/jox16060190

**Published:** 2026-10-07

**Authors:** Kumud Kant Awasthi, Vaibhav Sharma, Anuj Sharma, Garima Awasthi, Manpreet Kaur, Yogesh Kumar, Rajeev Kumar, Gaurav Kumar Singh, Mahipal Singh Sankhla, Saroj Kumar Amar

**Affiliations:** 1 School of Basic & Applied Sciences, K R Mangalam University, Gurugram 122003, Haryana, India; kkantawasthi@gmail.com; 2 Department of Medical Elementology and Toxicology, School of Chemical and Life Sciences, Jamia Hamdard, New Delhi 110062, Delhi, India; vaibhav.s4n6@gmail.com (V.S.); gariimaa21@gmail.com (G.A.); 3 Department of Forensic Science, Parul Institute of Applied Sciences, Parul University, Vadodara 391760, Gujarat, India; anujs0353@gmail.com; 4 School of Pharmaceutical Sciences, CGC University, Mohali 140307, Punjab, India; mksuprachem@gmail.com; 5 Department of Forensic Science, College of Allied and Healthcare Sciences, Teerthanker Mahaveer University, Moradabad 244001, Uttar Pradesh, India; yogesh.paramedical@tmu.ac.in; 6 Department of Forensic Science, IILM University, Greater Noida 201306, Uttar Pradesh, India; rajeev4n6@gmail.com; 7 Department of Forensic Science, Sharda School of Allied Health Sciences, Sharda University, Greater Noida 201306, Uttar Pradesh, India; grvsngh01@gmail.com; 8 Department of Forensic Science, School of Basic and Applied Science, Adamas University, Kolkata 700126, West Bengal, India

**Keywords:** nanotoxicology, mitochondria, epigenetics, biomarkers

## Abstract

Zinc oxide nanoparticles (ZnO NPs) rank among the highest-volume engineered nanomaterials, and their partial dissolution generates a composite particle and ion exposure that converges on three measurable molecular layers. This review consolidates quantitative endpoints reported across in vitro, rodent, avian, piscine and controlled human exposures and organises them into mitochondrial, redox and epigenetic signature classes. Reported responses include mitochondrial membrane potential loss to 55 percent of control, mitochondrial DNA copy number shifts spanning 290 percent to 80 percent of control, PGC-1 alpha suppression to 48.6 percent, global 5-methylcytosine reduction to 42 percent, histone G9a induction near 50-fold, and transcriptomic responses reaching 2703 differentially expressed genes. Cross-layer convergence is anchored by zinc homeostasis regulators, heme oxygenase 1, one carbon metabolite accumulation and ferroptotic lipid remodelling across species. A candidate integrated biomarker panel is proposed here together with the analytical and dosimetric constraints that presently limit its regulatory adoption.

## 1. Introduction

Zinc oxide nanoparticles occupy an unusual position among engineered nanomaterials because their toxicological behaviour is governed simultaneously by particulate and ionic determinants. ZnO is an ion-shedding oxide whose solubility in biological media releases Zn^2+^ at concentrations sufficient to saturate cellular buffering capacity, and the resulting perturbation cannot be attributed cleanly to either the intact particle or the dissolved cation [1,2]. Annual global production of ZnO NPs has been estimated at approximately one million tonnes, distributed across sunscreens, food contact materials, textile finishes, antimicrobial coatings, feed additives and semiconductor devices [3,4,5]. Nanoscale zinc oxide is deployed additionally as a seed priming agent in cereal agriculture [6], within nanoparticle-based interventions for metal(loid) stress in plants [7], and as a developing reagent in forensic latent fingerprint visualisation [8], widening the range of occupational settings in which exposure occurs. Quantum dot and related low-dimensional semiconductor nanostructures are under parallel development as electrocatalytic materials for hydrogen production [9], so the industrial settings in which nanoscale metal oxide and related semiconductor materials are synthesised and handled continue to widen. Occupational and consumer exposure follows inhalation, dermal, and oral routes, and controlled human inhalation of pure nano-sized ZnO produced concentration-dependent increases in symptoms, body temperature, acute phase proteins and circulating neutrophils at 1.0 mg/m^3^ and above, a level below the occupational exposure limit applied in many jurisdictions [10]. The demonstration of systemic inflammatory response in healthy volunteers at sublimit concentrations establishes that ZnO NP hazard characterisation cannot rest on cytotoxicity endpoints alone. Primary human nasal mucosa cells exposed in vitro display cytotoxic, genotoxic and pro-inflammatory responses at the portal of entry [11], and the molecular basis of ZnO NP genotoxicity has been reviewed as a distinct hazard class [12]. Mechanistic work has repeatedly identified zinc homeostasis disruption as the proximal event. Intracellular labile zinc rises after ZnO NP exposure, mitochondrial membrane potential collapses, and caspase-dependent death follows in a sequence reproduced across leukocyte, epithelial and neuronal models [13,14,15]. Subcytotoxic exposures give a more informative picture. At doses leaving viability unchanged, cells display metallothionein and zinc transporter induction, altered mitochondrial ultrastructure, autophagic vesicle accumulation and measurable epigenetic drift, with classical antioxidant enzyme genes remaining uninduced [15]. This dissociation between apical toxicity and molecular response is the central argument for signature-based hazard assessment. Neurotoxic mechanisms of ZnO NPs have been reviewed in depth [16], and machine learning-based toxicodynamic modelling is now being developed to predict metal nanoparticle hazard from physicochemical descriptors [17]. Omics platforms are applied across the inorganic nanoparticle class for precisely this purpose [18]. Epigenetic endpoints entered nanotoxicology as a distinct evidence stream approximately a decade ago [19]. DNA methylation, histone post-translational modification and noncoding RNA expression are sensitive to metal exposure, propagate through cell division, and can persist after the initiating exposure has cleared [20]. Genome-wide methylation profiling of nanomaterial-exposed cells has been proposed explicitly as a fingerprint assay resolving effects that conventional cytotoxicity and genotoxicity assays overlook [21]. Zinc is a structural cofactor of DNA methyltransferases, histone acetyltransferases, histone deacetylases and several histone demethylases, so zinc dyshomeostasis reaches the chromatin-modifying machinery independently of oxidative damage [22].

This review assembles quantitative endpoints from primary studies into three signature classes, mitochondrial, oxidative stress and epigenetic, and examines where the classes intersect at the level of omics-scale datasets. Only values explicitly reported in the cited source are tabulated. The objective is to define which measurements are sufficiently reproducible, dose anchored and mechanistically interpretable to serve as candidate biomarkers of ZnO NP exposure and effect.

## 2. Literature Search Strategy and Scope

This article is a critical narrative synthesis rather than a systematic review conducted under PRISMA 2020. No protocol was registered, and no formal risk of bias instrument was applied. The search was nevertheless structured, and its parameters are stated here so that the coverage of the evidence base can be judged. Records were identified in PubMed, Scopus and the Web of Science Core Collection, with forward and backward citation chaining from retrieved primary articles and from existing reviews of ZnO nanoparticle toxicology, genotoxicity and neurotoxicity [1,2,12,16]. Search strings combined material terms (“zinc oxide nanoparticle*”, “ZnO NP*”, “nano-ZnO”, “ZnO quantum dot*”) with endpoint terms (“mitochondri*”, “membrane potential”, “mitophagy”, “biogenesis”, “oxidative stress”, “reactive oxygen species”, “lipid peroxidation”, “DNA methylation”, “histone”, “microRNA”, “transcriptom*”, “proteom*”, “metabolom*”, “biomarker”) using Boolean AND and OR operators. The search was not restricted by publication year; the retrieved corpus contributing quantitative endpoints spans 2011 to 2026.

Records were retained where the study was a peer-reviewed primary experimental report in English, exposed a defined biological system to a ZnO nanomaterial, and reported at least one quantitative or explicitly directional mitochondrial, redox, epigenetic or omics-scale endpoint together with the exposure concentration and duration. Records were excluded where only apical cytotoxicity was reported without a molecular endpoint, where zinc was delivered exclusively as bulk or micron-scale material without a nanoscale arm, where ZnO formed part of a composite from which the zinc contribution could not be isolated, and where the record was a conference abstract, preprint or non-peer-reviewed report. Reviews were used for citation chaining and for contextual statements but contributed no quantitative entries to the data tables. Values were transcribed only as stated in the source, and a source reporting direction without a numeric value is marked as such in the corresponding table.

## 3. Physicochemical Determinants of Bioreactivity

### 3.1. Material, Dissolution and Uptake Descriptors

Signature interpretation is meaningless without dosimetric and material context. The ZnO NP preparations collated here span primary sizes from 5.4 nm to approximately 50 nm, hydrodynamic diameters from 83 nm to 2181 nm, and zeta potentials from −31.7 mV to +24.3 mV, with surface chemistry ranging from uncoated flame pyrolysis material to mandelic acid, gluconic acid, citric acid, folic acid, methoxyl, diethylene glycol and silica shell modifications [14,23,24]. Table 1 summarises the material and exposure descriptors of the studies contributing quantitative endpoints below.

Dissolution kinetics discriminate these preparations more sharply than primary size. Zn^2+^ released into complete culture medium over 24 h ranged from 1.29 mg/L for a mercaptopropyl trimethoxysilane core shell particle to 3.62 mg/L for a citric acid-modified particle, and dissolution had not reached equilibrium by 6 h in any preparation [14]. Matrix composition further modulates dissolution and fate, with food matrices altering both the dissolved fraction and the oral toxicity profile of ZnO NPs [5], and solubility ranks alongside particle identity as a determinant of the transcriptomic response of metal oxide nanoparticles [25]. Cell death in Jurkat cells at 25 mg/mL spanned 7 percent to 98 percent across the same particle set, and the rank order of cytotoxicity did not follow the rank order of Zn^2+^ release, indicating that surface ligand chemistry modulates rather than simply scales the relationship between dissolution and injury [14]. Chelation experiments reinforce this separation: in T24 bladder carcinoma cells; the zinc chelator TPEN did not alter viability at 1 to 10 mg/mL and only marginally reduced cytotoxicity at 15 to 25 mg/mL, yet it restored EZH2 and H3K27me3 levels in exposed cells, showing that ionic and particulate contributions dissociate at the level of individual molecular endpoints [26]. Extracellular versus intracellular dissolution is a further determinant. High-resolution transmission electron microscopy with energy-dispersive X-ray analysis of HepG2 cells exposed to subtoxic doses detected massive extracellular dissolution and intracellular accumulation of zinc ions with no evidence of intact particle entry alongside metallothionein 1X, ZnT1 and ZnT2 overexpression without induction of superoxide dismutase 1, superoxide dismutase 2 or catalase [15]. Contrasting observations exist in keratinocytes and bladder carcinoma cells, where electron microscopy localised particle aggregates in the perinuclear region and in the nucleus, respectively [26,27]. Uptake status is model-dependent and must be reported alongside any signature dataset. Surface functionalisation modifies cytotoxic potency independently of core size [28], and comparative ranking of ZnO nanoforms in A549 and J774 cells shows that potency is not predicted by nominal diameter alone [29].

**Table 1:** Material descriptors and exposure design of primary studies contributing quantitative endpoints to this review. DLS values are given as water/medium where both were reported. NR denotes a descriptor not reported in the cited source.

Material Descriptor	Model System	Dose Range	Duration	References
Uncoated flame pyrolysis ZnO, 14.7 ± 1.07 nm (TEM); DLS 159 ± 3/153 ± 4 nm; zeta −23.9 ± 0.2/−17.3 ± 0.1 mV	Human monocyte-derived macrophages, dendritic cells, Jurkat T cells	1 and 10 µg/mL	6 and 24 h	[14]
Diethylene glycol modified, 5.4 ± 0.5 nm; DLS 125 ± 4/278 ± 3 nm; zeta +19.2 ± 0.2/+13.6 ± 0.3 mV	Jurkat T cells	10 and 25 µg/mL	24 h	[14]
Folic acid-modified rod, 40.4 ± 2.6 nm diameter × 404 ± 8 nm length; DLS 412 ± 4/466 ± 3 nm; zeta −31.7 ± 0.3/−15.4 ± 0.5 mV	Jurkat T cells	10 and 25 µg/mL	24 h	[14]
Rod-shaped, 45 ± 27 nm (TEM); DLS 2181 ± 21/747 ± 25 nm; BET 35.34 ± 2.82 m^2^/g	Primary rat cortical astrocytes	4, 8, 12 µg/mL	6, 12, 24 h	[23]
Irregular spherical agglomerates; DLS 1387 ± 73 nm in water; charge reversal between water and medium	T24 human bladder carcinoma	1–25 µg/mL	48 h	[26]
Hexagonal prism, ~50 nm (TEM); DLS 500.8 nm; wurtzite by XRD	BV-2 murine microglia	5–30 µg/mL	4–24 h	[24]
Commercial ZnO nanopowder (Sigma 544906)	MRC5 human lung fibroblasts	0.125–8 µg/mL	24, 48, 72 h	[30]
ZnO quantum dots, 7.98 ± 0.31 nm (TEM); emission 566 nm	SMMC-7721 human hepatoma	10–90 µg/mL	24 h	[31]
Uncoated, 41.7 nm, 23.6 m^2^/g, purity 98.8%	Male Wistar rats, oropharyngeal aspiration	2, 4, 8 mg/kg once daily	3 days	[32]
Near spherical, ~30 nm, 50 m^2^/g, density 5.606 g/cm^3^	Laying hens, dietary administration	25, 50, 100 mg/kg	4 and 24 weeks	[33]
29.83 nm (XRD), 35 nm (SEM), hexagonal crystalline; LC50 72.48 ± 10.33 mg/kg body weight	Male Sprague Dawley rats, intraperitoneal	10, 20, 30 mg/kg on alternate days	21 days	[34]
Nominal ≤ 50 nm; DLS 214.16 ± 0.31 nm; zeta −16.11 ± 0.46 mV	Human iPSC-derived cardiomyocytes	10–500 µM	2–24 h	[3]
Nano-sized ZnO aerosol	16 healthy human volunteers, controlled inhalation with cycling workload	0.5, 1.0, 2.0 mg/m^3^	4 h per exposure day	[10]
ZnO NPs at subcytotoxic dose; no intact particle uptake detected by HRTEM–EDX	HepG2 human hepatocytes	subtoxic range	24 h	[15]
ZnO NPs, NR	A549 human alveolar epithelial cells	50 and 100 µg/mL	24 h	[35]
ZnO NPs, NR	Swiss mice, oral gavage	300 mg/kg	14 consecutive days	[36]
ZnO NPs < 100 nm	Pregnant rats; male offspring assessed at postnatal day 60	gestational, lactational or combined	gestation and lactation	[37]
ZnO NM110 reference material	PMA differentiated THP-1 macrophages	IC50 and IC50/4	4 h	[38]
ZnO NPs, NR	Rat NR8383 alveolar macrophages	one quarter IC50	4 h	[39]
ZnO NPs, NR	Mouse preimplantation embryos and embryonic stem cells	in vivo and in vitro exposure	preimplantation window	[40]

### 3.2. Harmonisation of Dose Descriptors

Comparison across the studies collated here is constrained less by the diversity of biological models than by the incompatibility of the descriptors used to express dose. Four metrics occur in the source literature: mass concentration in suspension (µg/mL), molar concentration (µM), mass per unit body weight or per unit of diet (mg/kg) and airborne mass concentration (mg/m^3^). These are interconvertible only where the reporting study also states molar mass, specific surface area, primary particle size and density. Table 2 sets out the conversions that the descriptors reported in each source permit.

Mass and molar concentration are related through the molar mass of ZnO (81.38 g/mol), so the 25, 50 and 100 µM exposures applied to human induced pluripotent stem cell-derived cardiomyocytes [3] correspond to 2.03, 4.07 and 8.14 µg/mL and fall within the 1 to 25 µg/mL band used by most of the in vitro studies collated here. Delivered surface area per unit volume follows from Equation (1), and particle number per unit volume from Equation (2) for approximately isometric particles:

S = Cm × SSA(1)

N = Cm/[ρ (π/6) d^3^] (2)

where Cm is the mass concentration, SSA the specific surface area, ρ the density of ZnO (5.606 g/cm^3^) and d the primary particle diameter.

Applied to the Brunauer–Emmett–Teller values that were reported, 12 µg/mL of the 45 nm rod used in primary astrocytes (35.34 m^2^/g) delivers 4.24 cm^2^/mL [23], the 8 mg/kg oropharyngeal dose of the 41.7 nm rat preparation (23.6 m^2^/g) delivers 0.189 m^2^ per kg body weight [32], and the 100 mg/kg dietary dose in laying hens (50 m^2^/g) delivers 5.0 m^2^ per kg of diet [33]. Where Brunauer–Emmett–Teller data were absent, the geometric specific surface area calculated from primary size and bulk density supplies a lower bound: 72.8 m^2^/g for the 14.7 nm uncoated material [14], 134.1 m^2^/g for the 7.98 nm quantum dot [31] and 21.4 m^2^/g for the preparations of approximately 50 nm [3,24]. The divergence between measured and geometric values is itself informative, since the laying hen material carries a reported specific surface area of 50 m^2^/g against a geometric value of 35.7 m^2^/g for a 30 nm sphere at bulk density, indicating surface roughness or porosity that diameter alone does not capture [33].

The consequence for interpretation is substantial. At a common nominal dose of 10 µg/mL, the 7.98 nm quantum dot delivers approximately 6.3 times the surface area and approximately 246 times the particle number of a 50 nm preparation, so endpoints recorded at matched mass are not recorded at matched interfacial or numerical dose. This is the quantitative expression of the particokinetic argument that equal mass concentrations of dissimilar nanomaterials do not constitute equal doses, and that sedimentation and diffusion govern the fraction of a nominal dose that reaches an adherent monolayer [41]. Deposited and retained particle surface area has been proposed as the descriptor that best aligns pulmonary responses across poorly soluble nanomaterials and as the basis of a dose concept for nanoparticle risk assessment [42], and for ZnO specifically, both mass and surface area have been evaluated as inhalation exposure metrics [43]. The qualification that applies to this material is that surface area performs less well for rapidly dissolving metal oxides because the reactive interface is lost once the particle dissolves within the phagolysosome, so the delivered ionic dose competes with the interfacial dose as the biologically effective descriptor [44].

Primary studies of ZnO nanomaterials should, accordingly, report mass concentration together with at least one interfacial descriptor, being either a measured Brunauer–Emmett–Teller specific surface area or a geometric surface area with the assumptions stated, the primary size distribution and density used for any conversion, the hydrodynamic size and zeta potential determined in the actual exposure medium, and the dissolution profile across the full exposure period rather than at a single early time point, since dissolution had not equilibrated by 6 h in any preparation examined systematically [14].

### 3.3. Physicochemical Descriptors Ranked by Influence on Signature Expression

Ranking the physicochemical descriptors by strength of influence is possible only within studies that varied one descriptor while holding the others constant, and on that restricted evidence base the order is dissolution behaviour, surface ligand chemistry, agglomeration state in the exposure medium, and primary size.

Dissolution behaviour ranks first because it sets the magnitude of the ionic term that drives zinc dyshomeostasis. Across a single well-characterised particle set, release into complete culture medium over 24 h spanned 1.29 to 3.62 mg/L, a 2.8-fold range, while cell death in Jurkat cells at 25 µg/mL spanned 7 to 98 percent [14]. Surface ligand chemistry ranks second because it dissociates dissolution from injury: within that same particle set, the rank order of cytotoxicity did not follow the rank order of Zn^2+^ release [14], and surface functionalisation alters cytotoxic potency independently of core size [28]. Agglomeration state in the exposure medium ranks third, since hydrodynamic diameters of 83 to 2181 nm were recorded for preparations whose primary sizes lay between 5.4 and 50 nm [14,23,26]; agglomerate size governs sedimentation, and therefore, the fraction of the nominal dose delivered to an adherent monolayer [41]. Primary size ranks last as an independent predictor, since comparative ranking of ZnO nanoforms in A549 and J774 cells showed that potency is not predicted by nominal diameter alone [29]; its influence is expressed largely through the surface area and dissolution rate that it determines.

Applied to the signature classes, endpoints demonstrably dependent on released zinc, such as enhancer of zeste homolog 2 expression and H3K27 trimethylation [26], would be expected to track dissolution most closely, while endpoints for which a particle-specific contribution has been demonstrated, such as regulation of embryonic development-associated microRNAs [45] and the plasma metabolite set that distinguishes ZnO nanoparticles from ZnSO_4_ at equal zinc dose [33], would be expected to track surface chemistry and agglomeration state. This partition has not been tested directly and is stated as a testable prediction rather than an established relationship.

### 3.4. Separating Particle-Specific from Ion-Driven Contributions

Because ZnO dissolves in biological media, an endpoint recorded after exposure describes a composite particle and ion exposure unless the design contains a control that isolates one term. Three designs achieve this. Chelation with N,N,N’,N’-tetrakis(2-pyridylmethyl)ethylenediamine (TPEN) removes labile zinc and identifies endpoints dependent on the ionic term. A ZnSO_4_ arm matched on intracellular rather than nominal zinc identifies endpoints that the ion alone reproduces and, by exclusion, those that require the particle. Direct determination of uptake and intracellular speciation establishes whether intact particles enter the cell at all.

Each design has been applied to ZnO, and the results do not converge on a single attribution. In T24 bladder carcinoma cells, TPEN did not alter viability at 1 to 10 µg/mL and reduced cytotoxicity only marginally at 15 to 25 µg/mL, yet it restored enhancer of zeste homolog 2 and H3K27 trimethylation and lowered RUNX3, assigning the polycomb axis to the ionic term while leaving the cytotoxic response unexplained by it [26]. In chicken ovarian granulosa cells, 5 µg/mL ZnO nanoparticles and 10 µg/mL ZnSO_4_ produced identical intracellular zinc concentrations and comparable growth inhibition, yet only the nanoparticle treatment regulated microRNAs associated with embryonic development, isolating a particle-specific transcriptional effect against a shared ionic effect on growth [45]. In laying hens dosed at equal zinc, 19 of 23 altered plasma metabolites differed between the nanoparticle and the salt [33]. In HepG2 cells, high-resolution transmission electron microscopy with energy-dispersive X-ray analysis detected extensive extracellular dissolution and intracellular zinc accumulation without evidence of intact particle entry, placing metallothionein 1X, ZnT1 and ZnT2 induction in that model on the ionic side [15]. Intracellular ion release has also been implicated in the histone H3 modifications produced by CuO and ZnO nanoparticles in human lung adenocarcinoma cells [46].

Table 3 assigns the endpoint classes compiled in this review to one of three attribution categories according to the control present in the source design. The tally is itself a finding. Ionic attribution has been tested for a minority of the endpoints collated here, and most of the quantitative values in Table 4, Table 5 and Table 6 therefore describe the response to a composite exposure and cannot be assigned to the nanoparticulate form specifically. Statements of particle specificity in this review are restricted to the studies carrying a matched zinc control [26,33,45] together with the intracellular ion release evidence [46]. Designs addressing this question should carry, as a minimum, a ZnSO_4_ arm matched on intracellular zinc and, where the endpoint is chromatin-associated, a chelator arm.

## 4. Mitochondrial Signatures

### 4.1. Membrane Potential, Permeability Transition and Intrinsic Apoptosis

**Figure 1:**
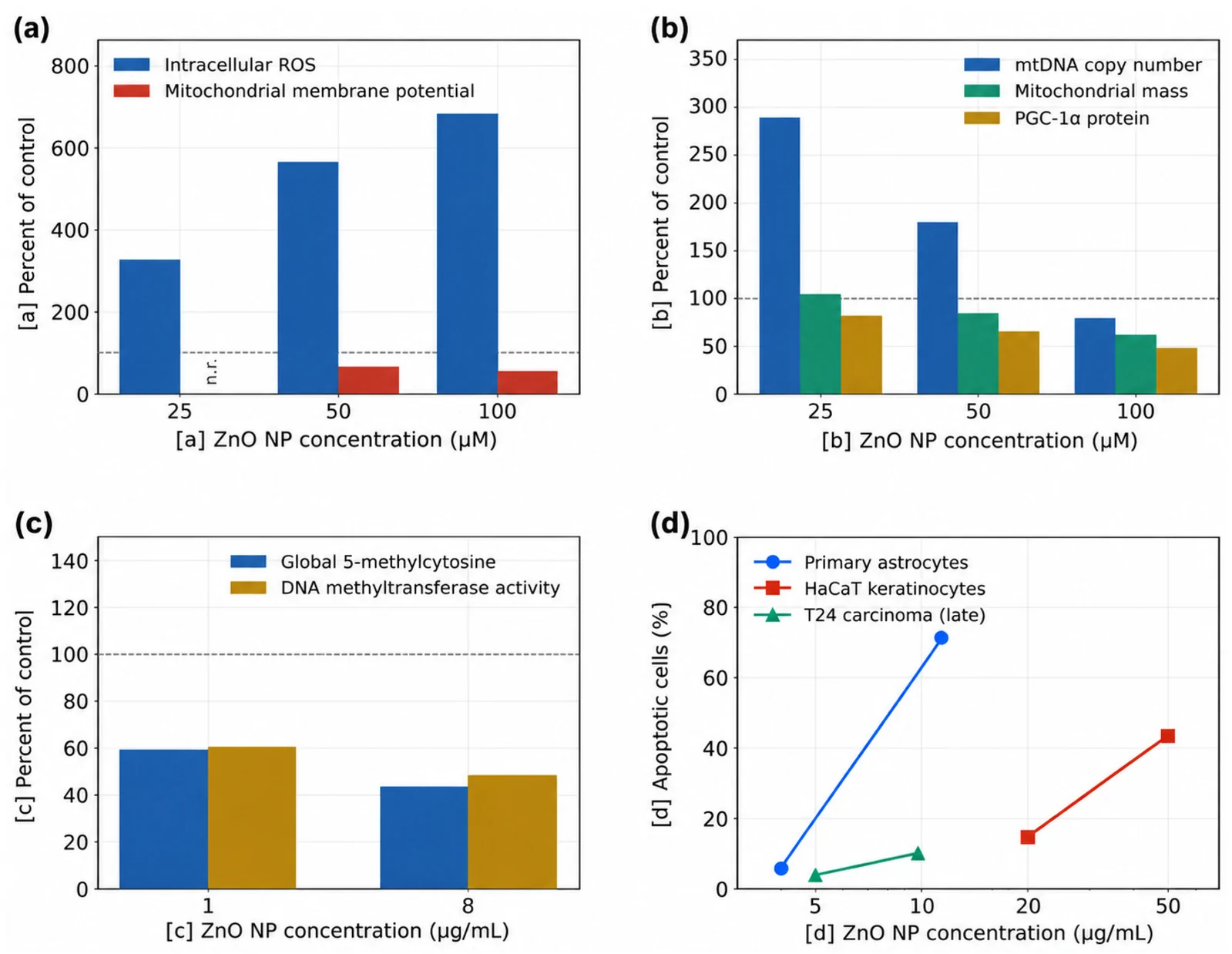
Quantitative endpoints replotted from values reported in the cited primary studies. (**a**) Intracellular reactive oxygen species and mitochondrial membrane potential in human iPSC-derived cardiomyocytes after 6 h [3]. (**b**) Mitochondrial DNA copy number, mitochondrial mass and PGC-1 alpha protein in the same model [3]. (**c**) Global 5-methylcytosine content and DNA methyltransferase activity in MRC5 lung fibroblasts at 24 h, discussed in Section 6.1 [30]. (**d**) Apoptotic cell fraction against exposure concentration in primary astrocytes at 6 h [23], HaCaT keratinocytes at 24 h [27] and T24 bladder carcinoma cells at 48 h [26]. Dashed lines mark the untreated control level. Variance data were not reported in numerical form in the primary sources for any of the endpoints plotted, so the values shown are single reported point estimates, and no error bars are given; they should not be interpreted as carrying an estimated precision. Mitochondrial membrane potential was not reported at 25 µM in the source study and is marked n.r. in panel (**a**).

Loss of inner membrane potential is the single most reproducible mitochondrial endpoint across ZnO NP studies and the one most amenable to quantitative expression. In human iPSC-derived cardiomyocytes, tetramethylrhodamine methyl ester fluorescence measured by high content analysis fell to 67 percent and 55 percent of control after 6 h exposure to 50 and 100 µM ZnO NPs at concentrations that produced no significant lactate dehydrogenase leakage [3]. Primary rat astrocytes displayed a dose-graded shift of JC-1 fluorescence from aggregate to monomer across 4 to 12 µg/mL, and the shift was reversed by 5 mM N acetylcysteine, placing the depolarisation downstream of reactive oxygen species generation [23]. In BV-2 microglia exposed to 10 µg/mL of 50 nm ZnO NPs, the JC-1 aggregate to monomer ratio declined progressively between 4 and 24 h [24]. HepG2 cells exposed to 14–20 µg/mL for 12 h showed reduced membrane potential together with an increased Bax to Bcl-2 ratio [54]. Rat H9c2 cardiomyoblasts reproduced the same redox and mitochondrial disturbance in a second cardiac model [56], ZnO NP exposure lowered intracellular ATP with concurrent mitochondrial damage and autophagic cell death [57], and ROS triggered mitochondria-mediated apoptosis after oxidative DNA damage was reproduced in zebrafish embryos [58]. Representative values from this and the following signature classes are plotted in Figure 1.

The apoptotic consequence scales steeply with dose. Annexin V and propidium iodide staining of astrocytes gave total apoptotic fractions of 5.79 percent at 4 mg/mL and 72.78 percent at 12 mg/mL after 6 h [23]. Keratinocytes progressed from 14.54 percent apoptosis at 20 mg/mL to 43.13 percent at 50 mg/mL over 24 h [27]. In T24 cells at deliberately subcytotoxic doses, the late apoptotic fraction rose from 1.27 percent in controls to 4.33 percent and 10.7 percent at 5 and 10 mg/mL over 48 h [26]. Transcriptional correlates in keratinocytes were pronounced, with Bax, Noxa and Puma messenger RNA rising approximately 10-fold, 15-fold and 300-fold at 50 mg/mL while Bcl-xl declined [27]. Oral coexposure to food grade titanium dioxide and ZnO NPs in rats induced mitochondrial permeability transition with cardiac damage, extending the endpoint to whole-animal exposure [52].

### 4.2. Respiratory Chain Function and Bioenergetic Capacity

Direct interrogation of the electron transport chain remains sparse. In A549 alveolar epithelial cells, 50 and 100 mg/mL ZnO NPs reduced complex I and complex III activity concentration dependently, with concurrent alteration of mitochondrial structure [35]. Nerve growth factor-differentiated PC12 cells subjected to extracellular flux analysis confirmed mitochondrial dysfunction at a noncytotoxic concentration that produced neurite shortening and degeneration, and integration of the accompanying microRNA and protein datasets nominated neurodegenerative disease and mitochondrial dysfunction pathways as the principal targets [59]. Quantitative proteomics of J774 macrophages identified a strong decrease in mitochondrial function with impaired phagocytosis and increased methylglyoxal-associated DNA damage, linking respiratory dysfunction to a genotoxic mechanism distinct from direct oxidant attack [51].

### 4.3. Biogenesis, Dynamics and Mitophagy

Mitochondrial biogenesis endpoints behave nonmonotonically and are, consequently, informative about dose regime. In iPSC-derived cardiomyocytes, mitochondrial DNA copy number rose to 290 percent and 180 percent of control at 25 and 50 µM ZnO NPs before falling to 80 percent at 100 µM, a biphasic profile consistent with compensatory replication under mild oxidative load followed by failure of that compensation [3]. Mitochondrial mass measured by MitoTracker Green tracked the collapse rather than the compensation, remaining at 105 percent of control at 25 µM and declining to 85 percent and 63 percent at 50 and 100 µM [3]. The transcriptional coactivator PGC-1 alpha declined to 82.1, 65.5 and 48.6 percent of control across the same concentration series, with its downstream effector TFAM reduced to 47.6 and 48.9 percent at 50 and 100 µM and cytochrome c oxidase subunit IV reduced by 24.5 and 38.1 percent [3]. A single biomarker of mitochondrial abundance is, therefore, inadequate; copy number and mass must be reported together with the PGC-1 alpha axis to permit correct interpretation.

Fission and fusion machinery respond in parallel. ZnO NP exposure of A549 cells increased the frequency of small rounded mitochondria and altered expression of the fission proteins Drp1 and Fis1 and the fusion proteins Mfn1, Mfn2 and OPA1 [35]. Selective clearance proceeds through the PINK1 and parkin axis. In BV-2 microglia, 10 mg/mL ZnO NPs elevated PINK1, reduced cytosolic parkin and increased mitochondrial parkin without changing the total parkin pool, indicating translocation rather than induction [24]. Knockdown of PINK1 abolished parkin recruitment, increased cleaved caspase 9 at every sampled time point and reduced survival relative to wild-type cells, establishing mitophagy as cytoprotective rather than executioner [24]. Mitophagic activation coupled to lipotoxicity has been described in freshwater teleost fish at environmentally relevant concentrations [60]. Autophagic flux is engaged more broadly, with ZnO NPs enhancing autophagy through PI3K, Akt and mTOR inhibition in murine peritoneal macrophages and thereby promoting apoptosis [61]. Subtoxic exposure of HepG2 cells produced abnormal mitochondrial morphology with autophagic vesicles, and a role was proposed for mitochondria in sequestering excess zinc that becomes lethal once storage capacity is exceeded [15]. In A549 lung epithelial cells, the autophagic response is harnessed to execute cell death rather than to protect [62], and in neuronal models, ZnO NPs drive ferroptotic death both in vitro and in vivo [63], indicating that the terminal death mode is model-dependent even where the upstream organelle lesion is shared.

## 5. Oxidative Stress Signatures

### 5.1. Reactive Oxygen Species and Thiol Redox Status In Vitro

Intracellular oxidant generation after ZnO NP exposure is concentration-dependent, kinetically transient in some models and absent at genuinely low dose in others. High content quantification in iPSC-derived cardiomyocytes recorded increases in CM-H2DCFDA fluorescence of 230, 467 and 582 percent of control at 25, 50 and 100 µM after 6 h [3]. HaCaT keratinocytes exposed to 20 µg/mL showed elevated fluorescence at 6 h that returned to control level by 24 h and remained at that level for 10 days, demonstrating that a single late time point will misclassify the redox status of this system [27]. Astrocytes showed dose-dependent increases at 8 and 12 µg/mL after 6 h with further elevation at 12 h, and pretreatment with 5 mM N acetylcysteine suppressed both the oxidant signal and the downstream JNK phosphorylation and PARP cleavage [23]. BV-2 microglia exposed to 10 µg/mL displayed a time-dependent rise between 4 and 12 h [24]. Human lung fibroblasts and *Drosophila melanogaster* exposed in parallel showed oxidative stress-dependent cytotoxicity and genotoxicity with Nrf2 pathway engagement [64], the same pathway activated in SK-N-SH neuroblastoma cells coexposed to ZnO NPs and a brominated flame retardant [65]. Spermatogonial cells [66] and human glial cells assessed alongside zebrafish embryos [67] extend the oxidant response to reproductive and neural targets.

The negative results are equally informative. T24 bladder carcinoma cells exposed to 5 and 10 µg/mL for 48 h showed no significant increase in total reactive oxygen species, no rise in extracellular 8-oxo-7,8-dihydro-2′-deoxyguanosine and no change in gamma H2AX, despite clear cell cycle arrest, apoptosis and histone methylation changes [26]. Subtoxic exposure of HepG2 cells did not induce superoxide dismutase 1, superoxide dismutase 2 or catalase transcripts, with only modest upregulation of heme oxygenase 1 and the glutamate cysteine ligase modifier subunit, both of which respond to metal loading as well as to oxidants [15]. Thiol depletion at low dose is similarly modest: total glutathione in MRC5 lung fibroblasts fell from 0.83 to 0.49 nmol/mg protein at 8 µg/mL after 24 h without reaching statistical significance, yet the same exposures produced substantial DNA methylation change [30]. Oxidative stress is, therefore, neither necessary nor sufficient for the full ZnO NP signature and should not be treated as the sole upstream node.

The operational consequence is that reactive oxygen species endpoints fail the test required of a standalone biomarker in both directions. They generate false negatives: T24 cells showed no increase in total reactive oxygen species, no rise in extracellular 8-oxo-7,8-dihydro-2′-deoxyguanosine and no change in gamma H2AX while displaying enhancer of zeste homolog 2 loss, H3K27 trimethylation depletion, cell cycle arrest and apoptosis [26], and HepG2 cells at subtoxic dose showed no induction of superoxide dismutase 1, superoxide dismutase 2 or catalase while displaying metallothionein and zinc transporter induction with altered mitochondrial ultrastructure [15]. They also generate false positives with respect to material attribution, since malondialdehyde elevation and antioxidant enzyme depletion of comparable magnitude follow exposure to graphene oxide and multiwalled carbon nanotubes in the same rodent models [68,69,70]. A third limitation is temporal: the keratinocyte oxidant signal peaked at 6 h and had returned to the control level by 24 h, where it remained for 10 days, while chromatin condensation and histone methylation changes measured at 24 h persisted [27]. A single late sampling point will record absence of oxidative stress in a system that experienced it.

Integration with the mitochondrial and epigenetic layers addresses each failure mode separately rather than generically. Mitochondrial endpoints supply sensitivity at subcytotoxic dose together with a defined mechanistic sequence, since membrane potential loss, biogenesis failure and PINK1-dependent mitophagy were recorded at concentrations producing no significant lactate dehydrogenase leakage and were functionally validated by knockdown [3,24]. Epigenetic endpoints supply persistence, since chromatin marks propagate through cell division and outlast the initiating exposure [20,27], and in one system, a chromatin-directed inhibitor rescued an apical developmental outcome [40]. Material discrimination is supplied instead by the zinc-specific exposure markers, metallothionein and the zinc transporters, which respond within 4 h and are not induced by carbon-based nanomaterials [14,15,38]. No single layer carries all three properties, which is the argument for a panel rather than for substituting one endpoint class for another.

### 5.2. Antioxidant Enzyme and Lipid Peroxidation Endpoints In Vivo

Whole-animal studies deliver the most consistent quantitative redox data. Oral administration of 50 mg/kg to rats for 14 days raised plasma aspartate aminotransferase 2.8-fold, alanine aminotransferase 2.7-fold and alkaline phosphatase 1.97-fold, increased hepatic malondialdehyde and reduced hepatic superoxide dismutase and glutathione peroxidase activity, with centrilobular necrosis and microvesicular steatosis on histology [49]. Intraperitoneal administration of 80 mg/kg three times weekly for four weeks raised serum aspartate aminotransferase by 41 percent and alanine aminotransferase by 18 percent, hepatic malondialdehyde by 38 percent and renal malondialdehyde by 67 percent, with BAX increased 47 percent, interleukin 1 beta 23 percent and tumour necrosis factor alpha 82 percent [50]. Subacute oral exposure of mice to 300 mg/kg for 14 consecutive days produced hepatic nanoparticle accumulation, elevated alanine aminotransferase and alkaline phosphatase, increased lipid peroxidation, a significant rise in formamidopyrimidine DNA glycosylase-sensitive lesions and positive terminal deoxynucleotidyl transferase dUTP nick end labelling [36]. Intraperitoneal dosing at 10, 20 and 30 mg/kg on alternate days for 21 days produced dose-dependent alteration of hepatic and renal oxidative stress enzymes against a median lethal dose of 72.48 plus or minus 10.33 mg/kg [34]. Renal endpoints follow the same direction, with superoxide dismutase decreased and malondialdehyde increased in podocytes and in rats dosed at 3 mg/kg per day for five days [71]. Oral administration in mice also produces reproductive organ dysfunction with altered apoptotic gene expression [72]; gestational exposure alters embryo-foetal development in rats [73]; and continuous dermal exposure of barrier-compromised mouse skin produces melanoma-like lesions through oxidative stress-activated NF-kappa B signalling [74]. Equivalent oxidant-driven organ injury is documented for carbon-based nanomaterials in the same rodent models, including graphene oxide hepatotoxicity [68], multiwalled carbon nanotube hepatotoxicity [69] and nanoscale graphene oxide effects on testes and fertility [70], indicating that the redox signature is shared across nanomaterial classes and is, therefore, of limited value for material attribution. In plants, biosynthesised ZnO NPs produce dose-dependent oxidative stress and growth inhibition in rice seedlings [75]. Developmental exposure extends the signature across generations: maternal administration during gestation, lactation or both reduced offspring brain weight, body weight and survival index and produced, at postnatal day 60, elevated 8-hydroxy-2′-deoxyguanosine, malondialdehyde and nitric oxide with depletion of glutathione, glutathione S transferase, glutathione peroxidase, superoxide dismutase and catalase [37].

### 5.3. Oxidative Nucleic Acid Lesions and Inflammatory Readouts

Oxidative DNA lesions are detectable by orthogonal methods. HepG2 cells exposed to 14 to 20 mg/mL for 12 h showed increased formamidopyrimidine DNA glycosylase sensitive sites in the comet assay [54]. Untargeted metabolomics of SMMC-7721 hepatoma cells gave an independent measure, with 8-hydroxy-deoxyguanosine elevated 6.71-fold at 20 mg/mL and 22.95-fold at 50 mg/mL ZnO quantum dots [31]. Keratinocytes showed gamma H2AX signal increase at 20 and 50 mg/mL after 24 h [27].

Inflammatory readouts accompany the redox response in vivo and in humans. Oropharyngeal aspiration of 2, 4 and 8 mg/kg in rats raised serum interleukin 8, interleukin 1 beta and tumour necrosis factor alpha; increased blood leukocyte, lymphocyte, monocyte and neutrophil counts; and increased bronchoalveolar lavage fluid protein and lactate dehydrogenase activity, with histology progressing from mild infiltration at 2 mg/kg to complete consolidation at 8 mg/kg [32]. Controlled human inhalation produced concentration-dependent increases in symptoms, body temperature, acute-phase proteins and blood neutrophils, with significant effects from 1.0 mg/m^3^ and inflammatory markers in blood identified as the most sensitive parameters [10]. Rat inhalation and intratracheal instillation give concordant pulmonary inflammatory profiles for 35 nm ZnO [76], the inflammatory signal is transduced through reactive oxygen species-dependent NF-kappa B-mediated NLRP3 inflammasome activation in alveolar epithelial cells [77], and an in vitro air–blood barrier model reproduces epithelial damage with endothelial activation [78]. Barrier models of the gastrointestinal tract show a parallel response after oral exposure [79], and ecological studies in fish and invertebrate models confirm that the same inflammatory and oxidant endpoints operate at environmentally relevant concentrations [80,81].

## 6. Epigenetic Signatures

### 6.1. DNA Methylation and the Methyltransferase Machinery

Global cytosine methylation is reduced by ZnO NP exposure in every mammalian system in which it has been measured, but the enzymatic route to that reduction differs between models, and this divergence is the principal unresolved question in the field.

In MRC5 human lung fibroblasts, enzyme-linked immunosorbent assay quantification of 5-methylcytosine gave 59 percent and 58.8 percent of control at 24 and 48 h for a sublethal 1 µg/mL exposure, and 43 percent and 42 percent at 8 µg/mL [30]. DNA methyltransferase activity measured colorimetrically was repressed by approximately 40 percent at 1 µg/mL after 24 h and fell to 48 percent of control at 8 µg/mL [30]. Transcript analysis showed relative expression of 0.63 to 0.69 for Dnmt1, 0.61 to 0.68 for Dnmt3B and 0.98 for Dnmt3A [30]. Hypomethylation in this model is attributable to loss of methyltransferase capacity, and it occurred at concentrations that produced no significant glutathione depletion and 80 percent viability, satisfying the operational definition of an early exposure marker.

HEK-293 cells exposed to ZnO NPs showed the same directional change in 5-methylcytosine but reached it by an opposite enzymatic route. Global 5-methylcytosine decreased while 5-hydroxymethylcytosine increased, with significant upregulation of ten eleven translocation methylcytosine dioxygenase genes and no upregulation of DNA methyltransferases [48]. Locus-resolved analysis at the LINE-1, subtelomeric D4Z4 and pericentromeric NBL2 repeat families and at the promoters of AOX1, HMOX1, NCF2 and SOD3 demonstrated that the response is not uniformly distributed across the genome [48]. Active TET-mediated demethylation and passive maintenance failure are mechanistically distinct and carry different implications for persistence, since the former is enzymatically driven and potentially reversible, while the latter propagates through replication. Cell type, exposure duration and dose regime all differ between the two studies, and no experiment has yet tested both routes in a single system.

Developmental exposure produces a coexisting global and locus-specific pattern. Offspring of rats exposed to ZnO NPs during gestation, lactation or both displayed global DNA hypomethylation together with targeted hypermethylation of the brain-derived neurotrophic factor promoter, and the promoter hypermethylation correlated with reduced brain-derived neurotrophic factor messenger RNA [37]. A global hypomethylation readout alone would have missed the functionally consequential locus. Comparable coupling of transcriptomic and epigenetic change has been recorded for copper(II) oxide nanoparticles after subchronic inhalation in mice [82], and multigenerational exposure of a soil invertebrate to tungsten carbide cobalt nanomaterials alters global DNA methylation across generations [83], establishing that the phenomenon is neither species- nor material-restricted.

Zinc dependence of the methylation machinery provides a mechanistic basis independent of oxidant chemistry, since DNA methyltransferases, histone acetyltransferases, histone deacetylases and several histone demethylases require zinc for catalytic activity or structural integrity [22]. One carbon metabolism supplies a second route. Untargeted metabolomics of ZnO quantum dot-exposed hepatoma cells recorded S-adenosylhomocysteine at 13.16-fold and 23.88-fold of control at 20 and 50 mg/mL [31]. S-adenosylhomocysteine is a product inhibitor of S-adenosylmethionine-dependent methyltransferases, and accumulation of this magnitude is a plausible nonenzymatic contributor to hypomethylation that no ZnO NP study has tested directly.

### 6.2. Histone Post-Translational Modifications

Histone marks respond at concentrations comparable to those affecting DNA methylation and show clear model dependence in direction.

HaCaT keratinocytes exposed for 24 h showed global H4K5 acetylation approximately 4-fold lower and H3K9 dimethylation approximately 1.5-fold higher with increasing ZnO NP concentration, accompanied by chromatin condensation in which the number of moderately condensed nuclei was nearly 3.5-fold higher than control [27]. The enzymatic correlates were unambiguous: at 50 µg/mL, the histone methyltransferase G9a transcript was nearly 50-fold higher than control and GLP nearly 5-fold higher, while the acetyltransferases GCN5, P300 and CBP were significantly downregulated [27]. Cell cycle distribution shifted to 33.55 percent of cells in G2/M against 16.65 percent in controls, with the S-phase fraction falling from 38.83 percent to 22.33 percent [27].

T24 bladder carcinoma cells exposed to 5 and 10 µg/mL for 48 h showed the opposite methylation direction at a different residue. Enhancer of zeste homolog 2 declined at both messenger RNA and protein levels, global H3K27 trimethylation decreased while total histone H3 remained constant, and chromatin immunoprecipitation with quantitative PCR showed reduced H3K27me3 occupancy at the RUNX3 promoter with corresponding increases in RUNX3 transcript and protein [26]. CBP, HDAC1 and LSD1 were essentially unchanged at 10 µg/mL, indicating selectivity for the polycomb axis rather than global disruption of chromatin enzymes [26]. Addition of the zinc chelator TPEN raised EZH2 and H3K27me3 and lowered RUNX3 in exposed cells, assigning a substantial share of the effect to released Zn^2+^ [26].

Preimplantation development provides the most mechanistically complete histone dataset. ZnO NPs impaired mouse preimplantation embryonic development in vivo and in vitro and reduced the pluripotency of mouse embryonic stem cells in teratoma and diploid chimera assays [40]. Integration of RNA sequencing, CUT&Tag and assay for transposase-accessible chromatin sequencing showed elevated H3K9 trimethylation, expanded heterochromatin and consequent repression of developmental and pluripotency genes, and the H3K9 methyltransferase inhibitor chaetocin reversed the embryotoxicity [40]. Pull-down and immunoprecipitation assays indicated direct interaction between ZnO NPs and H3K9me3 [40]. Chemical rescue of an apical developmental endpoint by a chromatin-directed inhibitor is the strongest available causal evidence that epigenetic modification is not merely correlated with ZnO NP toxicity.

### 6.3. Noncoding RNA

Small RNA responses are consistently detected, but the individual species reported vary with organism, tissue and platform, which limits their present value as generic biomarkers. Serum exosomes from rats given 4 mg/kg ZnO NPs by oropharyngeal aspiration contained 23 differentially expressed microRNAs at a threshold of 1.5-fold change and P not exceeding 0.05, comprising 16 upregulated and 7 downregulated species, of which 12 changed more than 5-fold [32]. Target prediction converging two databases assigned 2927 putative target genes to 14 of these microRNAs, and enrichment implicated response to toxic substances, cellular response to apoptosis, vesicle-mediated transport, oxidative stress, interleukin 1 signalling and autophagy [32]. The network was centred on Chst1, Nrbf2 and Scn9a, with miR-134-5p, miR-207 and miR-465-5p representing the upregulated set and miR-30b-5p, miR-19a-3p and miR-130a-3p the downregulated set [32]. Integrated messenger RNA and microRNA sequencing in differentiated THP-1 macrophages exposed at the maximum concentration producing no more than 15 percent cytotoxicity identified a coregulated cluster relevant to metal ion homeostasis, annotated as canonical or variant isoforms of hsa-miR-142-5p, hsa-miR-342-3p, hsa-miR-5100, hsa-miR-6087, hsa-miR-6894-3p and hsa-miR-7704 [84]. Hsa-miR-5100 was differentially expressed in response to silver, titanium dioxide and zinc oxide nanoparticles at both 6 and 24 h, making it the closest available candidate for a pan-metal-oxide microRNA marker, and the magnitude of microRNA change tracked the health hazard potential of the material [84]. Differentiation state and exposure form are both discriminated. Nerve growth factor differentiated PC12 cells showed deregulation of 15 microRNAs and 267 proteins at a noncytotoxic concentration against 8 microRNAs and 207 proteins in undifferentiated cells [59]. In chicken ovarian granulosa cells, 5 mg/mL ZnO NPs and 10 mg/mL ZnSO_4_ produced identical intracellular zinc concentrations and similar growth inhibition, yet only the nanoparticle treatment regulated microRNAs involved in embryonic development [45]. In mouse testes, ZnO NP exposure reduced miR-342-5p, which targets Erc1 and thereby restrains NF-kappa B signalling, and suppression of Erc1 rescued spermatocyte ferroptosis [55]. Maternal exposure raised brain miR-34a and lowered miR-29b in offspring at postnatal day 60 [37].

### 6.4. Reconciliation of Divergent Epigenetic Observations

Three directional conflicts run through the epigenetic evidence, and each admits a candidate resolution that the present literature does not yet test.

The first conflict concerns the enzymatic route to global hypomethylation. Loss of 5-methylcytosine proceeds through methyltransferase repression in MRC5 lung fibroblasts [30] and through ten eleven translocation dioxygenase induction in HEK-293 cells [48]. The two studies differ in cell identity, exposure duration and dose regime, and they differ also in oxidant status. Induction of the dioxygenases in the embryonic kidney model was attributed by its authors to nanoparticle-derived reactive oxygen species [48], whereas methyltransferase repression in the fibroblast model occurred at concentrations producing no significant glutathione depletion [30]. The two routes may accordingly correspond to different positions on the oxidant dose response rather than to irreconcilable cell type behaviour, with active demethylation engaged where oxidant load is sufficient to induce the dioxygenases and passive maintenance failure predominating below that threshold. Distinguishing these possibilities requires 5-methylcytosine, 5-hydroxymethylcytosine, methyltransferase activity, dioxygenase expression and oxidant status to be measured in the same samples across a common dose range, which no ZnO study has reported.

The second conflict concerns directional opposition in histone lysine methylation. Dimethylation and trimethylation at H3K9 increase in keratinocytes and in preimplantation embryos [27,40] while trimethylation at H3K27 decreases in bladder carcinoma cells [26]. The two marks are deposited by distinct enzyme families, G9a and GLP at H3K9 against polycomb repressive complex 2 at H3K27, and the bladder carcinoma study recorded CBP, HDAC1 and LSD1 as essentially unchanged, indicating selectivity for the polycomb axis rather than global disruption of chromatin enzymes [26]. Opposite directions at these two residues are not mutually exclusive observations, and the unresolved question is whether both axes move in opposite directions within a single cell type exposed to a single material. A third dataset reports histone H3 modification in human lung adenocarcinoma cells with intracellular ion release implicated [46], and cross-referencing the residues examined there against those in the keratinocyte, embryo and bladder datasets is the shortest available route to addressing that question.

The third conflict concerns the presence of a measurable oxidant signal in most models and its absence in bladder carcinoma cells showing clear chromatin change [26]. Dose regime and sampling time both differ. The bladder carcinoma exposures were deliberately subcytotoxic at 5 and 10 µg/mL and were sampled at 48 h, while the models reporting oxidant elevation used concentrations producing measurable viability loss and sampled between 4 and 12 h [3,23,24]. A transient oxidant excursion of the kind documented in keratinocytes, where the signal peaked at 6 h and had returned to control by 24 h [27], would not be detected at 48 h.

Resolution requires a design that the present literature does not contain. A single, fully characterised reference material would be applied at concentrations matched on both mass and surface area, sampled across a time course spanning the transient oxidant window and the persistent chromatin window, in a panel comprising at least one primary diploid fibroblast, one transformed epithelial line and one immune competent model, with paired ZnSO_4_ and chelator arms and with all three signature layers measured in the same samples. ZnO NM110 is already in regulatory circulation, has been used for transcriptomic profiling in differentiated THP-1 macrophages [38], and was ranked against other oxide nanomaterials in a nine-laboratory comparative programme [85], which makes it the obvious candidate material. Inter-laboratory formats for nanomaterial cytotoxicity and pro-inflammatory endpoints have been established in pan-European and North American consortia [86,87], so the operational infrastructure for such an exercise exists; what is absent is its extension to chromatin and mitochondrial endpoints.

## 7. Omics-Scale Datasets and Cross-Layer Convergence

### 7.1. Transcriptomic Response

Genome-wide profiling shows that the magnitude of the ZnO NP transcriptomic response is governed more by cell identity than by apical viability. Bead array profiling of three human immune-competent cell types exposed to 10 mg/mL uncoated ZnO NP for 24 h yielded 2703 differentially expressed genes in monocyte-derived macrophages, 980 in Jurkat T cells and only 12 in monocyte-derived dendritic cells, at a false discovery rate not exceeding 0.05 and absolute fold change of at least 1.5 [14]. Viability was approximately 55 percent in macrophages and approximately 70 percent in both dendritic and Jurkat cells, so an 80-fold difference in transcriptional response occurred at matched cytotoxicity [14]. Neither titanium dioxide nor 1 mg/mL ZnO NP produced any differentially expressed gene under the same thresholds, and metallothionein induction was the only response common to all three cell types [14].

Comparable studies reinforce the pattern. K562 chronic myeloid leukaemia cells yielded 429 differentially expressed genes at a fold change of at least four [88]. Rat NR8383 alveolar macrophages exposed for 4 h at one quarter of the median inhibitory concentration yielded 985 differentially expressed genes for ZnO [39]. Goat mammary epithelial cells showed more than 500 genes changed, with concurrent activation of extrinsic death receptor signalling through TNFα, TNFR1, FADD, caspase 8 and caspase 6; intrinsic signalling through Bax, cytochrome c, caspase 3 and caspase 9; and endoplasmic reticulum stress through PERK, ATF4, eIF2α and CHOP [53]. Reference material NM110 perturbed metal metabolism genes in THP-1 macrophages after only 4 h at both the median inhibitory concentration and one quarter of it [38]. In vivo, ZnO quantum dots produced 47 differentially expressed genes in mouse livers and 218 in lungs at a fold change above 1.5 and adjusted P below 0.05 [89], and oropharyngeal aspiration of 2, 10 or 50 mg nZnO produced the largest number of differentially expressed genes at day 1 together with the highest accumulation of F4/80 and CD3-positive cells [90]. Translational control is remodelled faster than transcription, with ribosome profiling of A549 cells resolving changes in the translatome within 1 h of exposure [91], and a toxicogenomic comparison across metal oxide nanoparticles of differing solubility identified a differentially expressed gene panel that tracked apical toxicity [25].

### 7.2. Proteomic and Metabolomic Response

Proteomic and metabolomic layers detect perturbation at doses below the transcriptomic and cytotoxic thresholds. In SMMC-7721 hepatoma cells, 20 µg/mL ZnO quantum dots produced no change in viability yet altered 174 metabolites and 105 proteins, comprising 64 upregulated and 41 downregulated species from 6469 identified proteins [31]. At 50 µg/mL, a concentration causing approximately 50 percent viability loss, 219 metabolites changed but only 8 proteins met the differential threshold, a divergence attributable to the collapse of coordinated protein regulation at overtly cytotoxic dose [31]. Enriched pathways common to both omics layers were alcoholism and Cushing syndrome, with purine metabolism and ferroptosis prominent among the metabolite pathways [31]. Mitochondrially annotated proteins upregulated at the noncytotoxic dose included cytochrome c oxidase copper chaperone COX17, mitochondrial thioredoxin and mitochondrial peptide chain release factor 1 [31]. Quantitative proteomics of J774 macrophages identified decreased mitochondrial function and phagocytosis with increased methylglyoxal-associated DNA damage [51]. Lipidomic profiling of kidney cells resolved specific aberrant lipid species and pathways not detectable in the transcriptomic layer [92]. The value and the current limitations of applying these platforms across the inorganic nanoparticle class have been reviewed systematically [18].

Metabolomic studies in vivo converge on energy metabolism. Rats given ZnO NPs for 14 days showed urinary and renal increases in taurine, lactate, acetate, creatine, phosphocholine, trimethylamine N oxide, alpha glucose and 3-D-hydroxybutyrate with decreases in lipid, succinate, citrate, alpha ketoglutarate, hippurate and 4-hydroxyphenylacetic acid, alongside raised serum creatinine and blood urea nitrogen and tubular epithelial necrosis at 1000 mg/kg [93]. Laying hens showed 23 altered plasma metabolites relative to control, of which 19 differed between ZnO NPs and ZnSO_4_ at equal zinc dose, with tricarboxylic acid cycle intermediates fumarate, citrate and succinate among them and hepatic steatosis developing over 24 weeks [33]. Rat lung and bronchoalveolar lavage fluid after inhalation showed increased isoleucine and valine and decreased acetate, trimethylamine N oxide, taurine, glycine, formate, ascorbate and glycerophosphocholine, with the dose response confined to the 250 nm preparation [94].

### 7.3. Nodes of Convergence

Four nodes recur across all three signature classes and across independent laboratories.

Zinc homeostasis is the first. Metallothionein induction is the only transcriptomic response shared by all cell types in a three-cell comparison [14]; metallothionein 1X, ZnT1 and ZnT2 are induced at subtoxic dose without antioxidant enzyme induction [15], metal metabolism genes are perturbed within 4 h [38], and metal ion homeostasis anchors the coregulated microRNA cluster in THP-1 cells [84]. Zinc dyshomeostasis is also the direct link to chromatin enzymes, which are zinc-dependent [22], and zinc chelation restores EZH2 and H3K27me3 in exposed bladder carcinoma cells [26]. Heme oxygenase 1 is the second. HMOX1 is upregulated in hepatocytes at subtoxic dose [15], and its promoter shows altered methylation in HEK-293 cells [48], placing one gene in both the redox and the epigenetic layer. One carbon and purine metabolism is the third. S-adenosylhomocysteine accumulation of 13- to 24-fold [31] provides a metabolic mechanism for methyltransferase inhibition operating independently of methyltransferase transcript loss [30] and of TET induction [48], and purine metabolism is the most consistently enriched metabolite pathway across doses [31]. Ferroptotic lipid remodelling is the fourth. ZnO NPs with ion-leaking properties disrupt reactive oxygen species and iron homeostasis to induce ferroptosis [47], ferroptosis is enriched among quantum dot-responsive metabolite pathways [31], aberrant lipid species are resolved by kidney cell lipidomics [92], mitophagy and lipotoxicity are coupled in fish [60], and in mouse testes ferroptosis is executed downstream of a microRNA change [55]. The last observation is the clearest documented instance of an epigenetic regulator sitting upstream of a redox-dependent death mechanism in ZnO NP toxicity.

## 8. Candidate Integrated Biomarker Panel

### 8.1. Panel Composition and Evidence Grading

A biomarker panel for ZnO NP hazard assessment must satisfy four criteria: measurable below the cytotoxic threshold, mechanistically interpretable, reproduced in at least two independent models, and quantifiable in an accessible matrix. Table 7 assembles the candidates meeting these criteria, graded by evidence status. The panel is deliberately layered. Mitochondrial membrane potential and mitochondrial DNA copy number report acute organelle status but behave nonmonotonically and require parallel measurement of the PGC-1 alpha axis [3]. Redox indices are the most widely measured and the least specific, since malondialdehyde, superoxide dismutase and glutathione peroxidase respond to almost any oxidant stressor; their value lies in dose anchoring rather than material attribution [34,49,50]. Metallothionein and zinc transporter induction shows the reverse profile, being specific to zinc loading and detectable within 4 h yet reporting exposure rather than adverse effect [14,15,38]. Epigenetic indices occupy the most useful position: detectable at sublethal dose [30,48], persistent through cell division [20], and in one system, reversible by a chromatin-directed inhibitor with rescue of an apical outcome [40]. Circulating exosomal microRNAs and the miR-5100 containing isomiR cluster are the only candidates presently measurable in a minimally invasive matrix [32,84]. Machine learning models trained on physicochemical descriptors and toxicodynamic endpoints offer a route to prioritising which of these analytes to measure for a given material [17].

### 8.2. Cross-Model Evidence, Practicality and Priority for Validation

Two statements require separation before the panel can be considered for use: which candidates are presently supported by reproduction across independent models and which rest on a single system.

Six candidates satisfy reproduction in three or more independent models. Mitochondrial membrane potential loss is recorded in cardiomyocytes, astrocytes, microglia, hepatocytes and mammary epithelial cells [3,23,24,53,54]. Global 5-methylcytosine reduction is recorded in lung fibroblasts, embryonic kidney cells and rat offspring brains [30,37,48]. Metallothionein and zinc transporter induction is recorded in immune-competent cells, hepatocytes and macrophages and is the earliest response detected [14,15,38]. Malondialdehyde elevation with antioxidant enzyme depletion is recorded across four or more in vivo studies [34,37,49,50]. Oxidative nucleic acid lesions are recorded by orthogonal platforms in cells, tissue and culture medium [31,36,37,54]. Blood acute-phase proteins and neutrophil counts are the only endpoints validated in controlled human exposure [10,95].

Eight candidates rest on a single model and require independent experimental validation before practical or regulatory application. These are the biphasic mitochondrial DNA copy number response together with the PGC-1 alpha, TFAM and cytochrome c oxidase subunit IV axis [3]; PINK1-dependent parkin translocation [24]; the methyltransferase repression route [30]; the dioxygenase and 5-hydroxymethylcytosine route [48]; G9a and GLP induction [27]; H3K27 trimethylation occupancy at the RUNX3 promoter [26]; the serum exosomal microRNA profile [32]; and S-adenosylhomocysteine accumulation [31]. Functional validation exists for two of them, by PINK1 knockdown [24] and by chaetocin rescue of embryotoxicity [40], which strengthens mechanistic interpretation without supplying cross-model replication.

Practical adoption depends additionally on matrix accessibility, platform cost and technical difficulty, which Table 8 sets out for each candidate analyte. On that basis the near-term ring trial set comprises the four analytes combining an accessible matrix, low technical burden and reproduction in three or more independent models: mitochondrial membrane potential by JC-1 or tetramethylrhodamine methyl ester, global 5-methylcytosine by colorimetric immunoassay, metallothionein and zinc transporter transcripts by reverse transcription quantitative PCR, and the routine blood inflammatory indices. Oxidative nucleic acid lesions are a reasonable fifth, with the qualification that platform harmonisation for 8-hydroxy-2′-deoxyguanosine measurement is itself unresolved. Chromatin immunoprecipitation resolved occupancy, mitochondrial protein axis measurement and small RNA sequencing carry high technical burdens with single-model support and are retained as mechanistic rather than regulatory endpoints. Standards development for transcriptomic and metabolomic in vitro methods entering regulatory toxicology is proceeding independently [96], and the epigenetic endpoints above will need to conform to it before entering a validated testing scheme.

## 9. Translational Relevance to Human Exposure

The mechanistic evidence compiled above derives overwhelmingly from cell lines, rodents, birds and fish, while the human evidence specific to ZnO is confined to controlled inhalation of pure nano-sized material in healthy volunteers [10,95]. Three observations bound the translational claim that the present data support.

The first is that the human evidence establishes hazard below the occupational limit but does not reach the molecular layers reviewed here. Controlled inhalation produced concentration-dependent increases in symptoms, body temperature, acute-phase proteins and blood neutrophils, with significant effects from 1.0 mg/m^3^ [10], and a subsequent controlled comparison of micro- and nano-sized ZnO recorded increases in neutrophils, monocytes and acute-phase proteins 22 h after exposure with no effect at 0.5 mg/m^3^ [95]. The human endpoint set is inflammatory and acute; no mitochondrial or chromatin endpoint has been measured in an exposed human.

The second is that molecular endpoints of the class proposed here have already been measured in workers exposed to other engineered nanomaterials, which establishes technical feasibility rather than ZnO-specific validity. Global DNA methylation has been quantified alongside oxidative stress markers in workers exposed to metal oxide nanomaterials [99], genome-wide methylation profiles have been reported for nanocomposite exposed workers and followed in the same individuals across four consecutive years [97,98], exhaled breath condensate and urinary biomarker panels have been applied in a multi-country nanomaterial worker cohort [100], and single-particle inductively coupled plasma mass spectrometry has been piloted for biomonitoring of metal engineered nanoparticles in worker matrices [101]. Human peripheral blood mononuclear cells exposed to ZnO nanoparticles in vitro show mitochondrial dysfunction and genotoxicity [102], so a single accessible matrix can, in principle, carry both the exposure and the effect arm of a biomonitoring design.

The third is that the candidate analytes divide sharply by matrix accessibility. Translatable to human population study at present are the blood inflammatory indices validated in controlled exposure [10,95], urinary and exhaled breath condensate oxidative lesion markers for which occupational protocols exist [99,100], global and repeat element methylation in peripheral blood leukocytes [97], and circulating microRNA profiles subject to prior analytical standardisation [32,84]. Restricted to pre-clinical use are mitochondrial membrane potential, mitochondrial DNA copy number in target tissue, the PGC-1 alpha and TFAM protein axis, PINK1-dependent parkin translocation, locus-resolved histone occupancy and promoter-specific methylation such as that at the brain-derived neurotrophic factor locus [3,24,26,37]. Each of these requires tissue that a workplace study cannot obtain, rests on a single model, or both.

Four challenges bound any such study. Workplace exposure to ZnO is rarely isolated, since zinc processing and galvanising generate mixed metal fumes, and attribution of an effect to ZnO specifically is correspondingly difficult [99]. No exposure biomarker discriminates nanoparticulate from ionic or dietary zinc, because metallothionein and the zinc transporters respond to zinc irrespective of its form [14,15]. Baseline methylation varies between individuals to a degree documented by repeated sampling of the same workers across four years, which sets the effect size that a cross-sectional design must exceed [98]. Smoking, age and co-exposure confound both the methylation and the oxidative lesion endpoints. The claim supportable on present evidence is, accordingly, laboratory feasibility of the full panel and operational feasibility of its blood-accessible subset, and not validated applicability to occupational risk assessment.

## 10. Methodological Constraints and Evidence Gaps

Five constraints limit immediate translation, and each is developed in the section indicated.

Dose metrics are not harmonised. Mass per volume, molarity, mass per body weight and airborne concentration are all in use; the same nominal mass corresponds to different particle numbers and surface areas depending on primary size and agglomeration state; and dissolution had not equilibrated by 6 h in any preparation examined systematically, so nominal dose is a poor surrogate for delivered zinc (Section 3.2 and Table 2) [14,23,24].

Particle and ion contributions are separated in only a minority of designs. Datasets generated without a chelator arm, a zinc salt arm matched on intracellular zinc, or determination of intracellular speciation describe a composite particle and ion exposure and cannot be attributed to the nanoparticulate form (Section 3.4 and Table 3) [26,33,45].

Directional conflicts remain unresolved. The enzymatic route to global hypomethylation, the direction of histone lysine methylation and the presence or absence of an oxidant signal all differ between models, and no study has applied a common material, dose metric and time course across the cell types concerned (Section 6.4) [26,27,30,40,48].

Temporal resolution is inadequate. The oxidant signal in keratinocytes peaked at 6 h and had returned to baseline by 24 h while chromatin changes persisted [27], and mitochondrial DNA copy number reversed direction between 50 and 100 micromolar within a single 6 h exposure [3]. Single-time-point designs will misassign both magnitude and direction of the response.

Human data are confined to acute inflammatory endpoints. No mitochondrial or epigenetic endpoint has been measured in an exposed human cohort, and the entire epigenetic evidence base rests on cell lines, rodents, birds and fish (Section 9) [10]. Peripheral blood mononuclear cell methylation and circulating exosomal microRNA profiling in exposed workers remains the most informative experiment the field has yet to perform. Pairing such biomonitoring with field-deployable nanosensor platforms of the type developed for aquatic metal ion detection would supply the external exposure term that current signature datasets lack [103].

## 11. Conclusions and Research Roadmap

ZnO nanoparticle toxicity resolves into three quantifiable molecular signature classes of differing maturity. Mitochondrial endpoints are the most mechanistically complete, with membrane potential loss to 55 percent of control, biphasic mitochondrial DNA copy number, PGC-1 alpha suppression to 48.6 percent and functionally validated PINK1-dependent mitophagy forming a coherent sequence. Oxidative endpoints are the most widely reproduced in vivo and the least specific, and their absence in at least one model showing clear chromatin change removes reactive oxygen species from the position of obligatory upstream trigger. Epigenetic endpoints are the most promising and the least mature: global 5-methylcytosine falls to 42 percent of control at sublethal dose, G9a transcript rises approximately 50-fold, H3K27me3 occupancy at a tumour suppressor promoter is zinc chelator-reversible, and inhibition of H3K9 trimethylation rescues an apical developmental outcome. Zinc homeostasis, heme oxygenase 1, S-adenosylhomocysteine accumulation and ferroptotic lipid remodelling connect the three classes into a single mechanistic architecture. The proposed panel is ready for cross-laboratory ring testing under harmonised dose metrics and paired ionic controls, and its extension to exposed human cohorts is the decisive step toward regulatory utility.

Five experimental requirements define the shortest route from the present evidence base to a validated panel. The first is standardised material characterisation reporting primary size distribution; measured specific surface area, density, hydrodynamic size and zeta potential in the actual exposure medium; and dissolution across the full exposure period rather than at a single early time point. The second is harmonised dosimetry, reporting mass concentration together with surface area and, for isometric materials, particle number, with delivered rather than nominal dose modelled for in vitro systems [41,42]. The third is time-resolved measurement spanning at least the 4 to 48 h interval, since the oxidant and chromatin responses occupy different windows [27]. The fourth is replication in independent models with paired ionic controls, a ZnSO_4_ arm matched on intracellular zinc and, for chromatin endpoints, a chelator arm [26,45]. The fifth is integrated measurement of transcriptomic, metabolomic, epigenomic and mitochondrial endpoints in the same samples rather than across separate experiments, using a common reference material such as ZnO NM110 [38,85] within an inter-laboratory format of the type already established for cytotoxicity and inflammatory endpoints [86,87]. Protocols for methylation and oxidative stress biomonitoring in workers exposed to other engineered nanomaterials already exist [97,99,100] and supply the operational template for the extension to exposed human cohorts.

## Figures and Tables

**Table 2:** Interconversion of dose descriptors for the studies contributing quantitative endpoints to this review. Mass and molar concentration are related through the molar mass of ZnO (81.38 g/mol). Surface area and particle number doses were calculated in the present review from the material descriptors reported in the cited source using Equations (1) and (2); they were not reported by the original authors. Geometric specific surface area (geo) assumes isometric particles at the bulk ZnO density of 5.606 g/cm^3^ and the reported primary diameter and is a lower bound for porous or rough material. BET denotes a measured Brunauer–Emmett–Teller value. NC, not calculable from the descriptors reported; NR, not reported.

Model and Reference	Reported Dose	Mass Equivalent	Specific Surface Area (m^2^/g)	Surface Area Dose	Particle Number Dose
hiPSC-derived cardiomyocytes [3]	25 µM	2.03 µg/mL	21.4 (geo, 50 nm)	0.43 cm^2^/mL	5.5 × 10^9^ per mL
hiPSC-derived cardiomyocytes [3]	50 µM	4.07 µg/mL	21.4 (geo, 50 nm)	0.87 cm^2^/mL	1.1 × 10^10^ per mL
hiPSC-derived cardiomyocytes [3]	100 µM	8.14 µg/mL	21.4 (geo, 50 nm)	1.74 cm^2^/mL	2.2 × 10^10^ per mL
Macrophages, dendritic cells, Jurkat T cells [14]	1 µg/mL	1 µg/mL	72.8 (geo, 14.7 nm)	0.73 cm^2^/mL	1.1 × 10^11^ per mL
Macrophages, dendritic cells, Jurkat T cells [14]	10 µg/mL	10 µg/mL	72.8 (geo, 14.7 nm)	7.28 cm^2^/mL	1.1 × 10^12^ per mL
Primary rat cortical astrocytes [23]	4 µg/mL	4 µg/mL	35.34 (BET)	1.41 cm^2^/mL	NC (rod shaped)
Primary rat cortical astrocytes [23]	8 µg/mL	8 µg/mL	35.34 (BET)	2.83 cm^2^/mL	NC (rod shaped)
Primary rat cortical astrocytes [23]	12 µg/mL	12 µg/mL	35.34 (BET)	4.24 cm^2^/mL	NC (rod shaped)
BV-2 murine microglia [24]	5 µg/mL	5 µg/mL	21.4 (geo, 50 nm)	1.07 cm^2^/mL	1.4 × 10^10^ per mL
BV-2 murine microglia [24]	10 µg/mL	10 µg/mL	21.4 (geo, 50 nm)	2.14 cm^2^/mL	2.7 × 10^10^ per mL
BV-2 murine microglia [24]	30 µg/mL	30 µg/mL	21.4 (geo, 50 nm)	6.42 cm^2^/mL	8.2 × 10^10^ per mL
SMMC-7721 hepatoma, ZnO quantum dots [31]	20 µg/mL	20 µg/mL	134.1 (geo, 7.98 nm)	26.8 cm^2^/mL	1.3 × 10^13^ per mL
SMMC-7721 hepatoma, ZnO quantum dots [31]	50 µg/mL	50 µg/mL	134.1 (geo, 7.98 nm)	67.1 cm^2^/mL	3.4 × 10^13^ per mL
MRC5 lung fibroblasts [30]	1 µg/mL	1 µg/mL	NR	NC	NC
MRC5 lung fibroblasts [30]	8 µg/mL	8 µg/mL	NR	NC	NC
T24 bladder carcinoma [26]	5 µg/mL	5 µg/mL	NR	NC	NC
T24 bladder carcinoma [26]	10 µg/mL	10 µg/mL	NR	NC	NC
Wistar rats, oropharyngeal aspiration [32]	2 mg/kg bw	2 mg/kg bw	23.6 (BET)	0.047 m^2^/kg bw	9.4 × 10^12^ per kg bw
Wistar rats, oropharyngeal aspiration [32]	4 mg/kg bw	4 mg/kg bw	23.6 (BET)	0.094 m^2^/kg bw	1.9 × 10^13^ per kg bw
Wistar rats, oropharyngeal aspiration [32]	8 mg/kg bw	8 mg/kg bw	23.6 (BET)	0.189 m^2^/kg bw	3.8 × 10^13^ per kg bw
Laying hens, dietary administration [33]	25 mg/kg diet	25 mg/kg diet	50 (BET)	1.25 m^2^/kg diet	3.2 × 10^14^ per kg diet
Laying hens, dietary administration [33]	50 mg/kg diet	50 mg/kg diet	50 (BET)	2.50 m^2^/kg diet	6.3 × 10^14^ per kg diet
Laying hens, dietary administration [33]	100 mg/kg diet	100 mg/kg diet	50 (BET)	5.00 m^2^/kg diet	1.3 × 10^15^ per kg diet
Sprague Dawley rats, intraperitoneal [34]	10 mg/kg bw	10 mg/kg bw	35.9 (geo, 29.83 nm)	0.359 m^2^/kg bw	1.3 × 10^14^ per kg bw
Sprague Dawley rats, intraperitoneal [34]	20 mg/kg bw	20 mg/kg bw	35.9 (geo, 29.83 nm)	0.718 m^2^/kg bw	2.6 × 10^14^ per kg bw
Sprague Dawley rats, intraperitoneal [34]	30 mg/kg bw	30 mg/kg bw	35.9 (geo, 29.83 nm)	1.077 m^2^/kg bw	3.9 × 10^14^ per kg bw
Human volunteers, controlled inhalation [10]	0.5, 1.0, 2.0 mg/m^3^	airborne mass concentration	NR	NC; conversion requires minute ventilation and regional deposition fraction	NC

**Table 3:** Attribution of compiled endpoints to ion-dependent, particle-specific or unattributed status according to the control present in the source design. Unattributed denotes an endpoint generated without a chelator arm, a matched zinc salt arm or determination of intracellular speciation; it does not imply that the endpoint is absent or unreliable.

Endpoint	Model	Attribution Control in the Source Design	Attribution	References
EZH2 protein and mRNA, global H3K27me3	T24 bladder carcinoma	3 µM TPEN co-exposure	Ion-dependent	[26]
RUNX3 derepression	T24 bladder carcinoma	3 µM TPEN co-exposure	Ion-dependent	[26]
Viability at 1 to 10 µg/mL	T24 bladder carcinoma	TPEN; viability unchanged	Not ion-dependent; particle contribution indicated	[26]
Embryonic development-associated microRNAs	Chicken ovarian granulosa cells	ZnSO_4_ at matched intracellular zinc	Particle-specific	[45]
Growth inhibition	Chicken ovarian granulosa cells	ZnSO_4_ at matched intracellular zinc	Ion-dependent; reproduced by the salt	[45]
Plasma metabolite profile (19 of 23 metabolites)	Laying hens	ZnSO_4_ at equal zinc dose	Particle-specific	[33]
Metallothionein 1X, ZnT1, ZnT2 induction	HepG2 hepatocytes	HRTEM–EDX; no intact particle uptake	Ion-driven	[15]
SOD1, SOD2, CAT noninduction	HepG2 hepatocytes	HRTEM–EDX; no intact particle uptake	Ion-driven	[15]
Histone H3 modification	Human lung adenocarcinoma cells	Intracellular ion release demonstrated	Ion-dependent	[46]
Ferroptosis induction	Ion-leaking ZnO preparations	Ion leaking property characterised	Ion-dependent	[47]
Cytotoxicity rank order across particle set	Jurkat T cells	Zn^2+^ release quantified per preparation	Not explained by ion release alone	[14]
Mitochondrial membrane potential loss	hiPSC-derived cardiomyocytes	None	Unattributed	[3]
mtDNA copy number, PGC-1 alpha, TFAM	hiPSC-derived cardiomyocytes	None	Unattributed	[3]
Respiratory complex I and III activity	A549 alveolar epithelial cells	None	Unattributed	[35]
PINK1 and mitochondrial parkin translocation	BV-2 microglia	None	Unattributed	[24]
Global 5-methylcytosine, DNMT activity	MRC5 lung fibroblasts	None	Unattributed	[30]
5-hydroxymethylcytosine, TET induction	HEK-293 cells	None	Unattributed	[48]
G9a and GLP induction, H4K5 acetylation loss	HaCaT keratinocytes	None	Unattributed	[27]
H3K9me3 elevation and embryotoxicity	Mouse preimplantation embryos	Chaetocin rescue; chromatin rather than ionic	Unattributed with respect to ion against particle	[40]
Serum exosomal microRNA profile	Wistar rats	None	Unattributed	[32]
Hepatic and renal MDA, SOD, GPx	Rats, oral and intraperitoneal	None	Unattributed	[34,49,50]
Brain 8-OHdG and BDNF promoter methylation	Rat offspring	None	Unattributed	[37]
Acute-phase proteins and blood neutrophils	Human volunteers	None	Unattributed	[10]
Transcriptomic response magnitude	Macrophages, dendritic cells, Jurkat	TiO_2_ comparator only	Unattributed with respect to ion against particle	[14]
S-adenosylhomocysteine accumulation	SMMC-7721 hepatoma	None	Unattributed	[31]

**Table 4:** Quantitative mitochondrial endpoints reported after ZnO nanomaterial exposure. Values are as stated in the cited source; qualitative entries are marked where the source reported direction without a numeric value.

Endpoint	Model	Exposure	Reported Value	References
Mitochondrial membrane potential (TMRM)	hiPSC-derived cardiomyocytes	50 µM, 6 h	67% of control	[3]
Mitochondrial membrane potential (TMRM)	hiPSC-derived cardiomyocytes	100 µM, 6 h	55% of control	[3]
Mitochondrial mass (MitoTracker Green)	hiPSC-derived cardiomyocytes	25/50/100 µM, 6 h	105%, 85%, 63% of control	[3]
mtDNA copy number (ND1/GAPDH)	hiPSC-derived cardiomyocytes	25/50/100 µM, 6 h	290%, 180%, 80% of control	[3]
PGC-1 alpha protein	hiPSC-derived cardiomyocytes	25/50/100 µM, 6 h	82.1%, 65.5%, 48.6% of control	[3]
TFAM protein	hiPSC-derived cardiomyocytes	50 and 100 µM, 6 h	47.6% and 48.9% of control	[3]
Spontaneous beat rate (microelectrode array)	hiPSC-derived cardiomyocytes	50 µM, 6 h	decreased 22.7% (55.11 ± 2.51 to 42.5 beats/min)	[3]
Total apoptotic fraction (Annexin V/PI)	Primary rat astrocytes	4 to 12 µg/mL, 6 h	5.79% to 72.78%	[23]
Mitochondrial membrane potential (JC-1)	Primary rat astrocytes	4–12 µg/mL, 6 h	dose-graded decrease, reversed by 5 mM N acetylcysteine	[23]
Bax, Bcl-2, cleaved caspase 3, cleaved PARP	Primary rat astrocytes	4–12 µg/mL, 6 h	Bax, cleaved caspase 3 and cleaved PARP increased; Bcl-2 decreased	[23]
Total apoptotic fraction (Annexin V/PI)	HaCaT keratinocytes	20 and 50 µg/mL, 24 h	14.54% and 43.13%	[27]
Bax, Noxa, Puma mRNA	HaCaT keratinocytes	50 µg/mL, 24 h	~10-fold, ~15-fold, ~300-fold increase	[27]
Late apoptotic fraction (Annexin V+/PI+)	T24 bladder carcinoma	0, 5, 10 µg/mL, 48 h	1.27%, 4.33%, 10.7%	[26]
Respiratory complex I and complex III activity	A549 alveolar epithelial cells	50 and 100 µg/mL	concentration-dependent reduction	[35]
Drp1, Fis1, Mfn1, Mfn2, OPA1 expression	A549 alveolar epithelial cells	50 and 100 µg/mL	fission and fusion protein expression altered; small round mitochondria increased	[35]
PINK1, cytosolic and mitochondrial parkin	BV-2 microglia	10 µg/mL, 4–24 h	PINK1 increased; cytosolic parkin decreased; mitochondrial parkin increased; total parkin unchanged	[24]
Cleaved caspase 9 after PINK1 knockdown	BV-2 microglia	10 µg/mL, 4–24 h	higher than wild type at every time point	[24]
Mitochondrial function (quantitative proteomics)	J774 murine macrophages	nonlethal dose	strong decrease in mitochondrial function; increased methylglyoxal-associated DNA damage	[51]
Mitochondrial permeability transition	Sprague Dawley rat heart, oral exposure	E171 and ZnO NP coexposure	permeability transition and cardiac damage induced	[52]
MMP, Bax, cytochrome c, caspase 3 and 9, Bcl2	Goat mammary epithelial cells	graded dose	MMP decreased; Bax, cytochrome c, caspase 3 and 9 increased; Bcl2 decreased	[53]

**Table 5:** Quantitative oxidative stress and inflammatory endpoints reported after ZnO nanomaterial exposure.

Endpoint	Model	Exposure	Reported Value	References
Intracellular ROS (CM-H2DCFDA)	hiPSC-derived cardiomyocytes	25/50/100 µM, 6 h	increased by 230%, 467%, 582%	[3]
Intracellular ROS (DCF)	Primary rat astrocytes	8 and 12 µg/mL, 6 and 12 h	dose- and time-dependent increase, abolished by 5 mM N acetylcysteine	[23]
Intracellular ROS (DCFH-DA)	BV-2 microglia	10 µg/mL, 4–12 h	time-dependent increase	[24]
Total ROS, 8-oxo-G and gamma H2AX	T24 bladder carcinoma	5 and 10 µg/mL, 48 h	no significant change from control	[26]
SOD1, SOD2, CAT transcripts	HepG2 hepatocytes	subtoxic dose	not induced; HMOX1 and GCLM slightly upregulated	[15]
Fpg-sensitive DNA sites (comet assay)	HepG2 hepatocytes	14–20 µg/mL, 12 h	significant increase	[54]
8-hydroxy-deoxyguanosine (untargeted metabolomics)	SMMC-7721 hepatoma, ZnO quantum dots	20 and 50 µg/mL, 24 h	6.71-fold and 22.95-fold of control	[31]
gamma H2AX (immunostaining and immunoblot)	HaCaT keratinocytes	20 and 50 µg/mL, 24 h	significant increase, greater at 50 µg/mL	[27]
Plasma AST, ALT, ALP	Rats, oral 50 mg/kg	14 days	2.8-fold, 2.7-fold, 1.97-fold increase	[49]
Serum AST and ALT	Rats, intraperitoneal 80 µg/kg	3 times weekly, 4 weeks	increased by 41% and 18%	[50]
Hepatic and renal MDA	Rats, intraperitoneal 80 µg/kg	3 times weekly, 4 weeks	increased by 38% and 67%	[50]
BAX, interleukin 1 beta, TNF alpha	Rats, intraperitoneal 80 µg/kg	3 times weekly, 4 weeks	increased by 47%, 23% and 82%	[50]
Hepatic lipid peroxidation and Fpg lesions	Mice, oral 300 mg/kg	14 consecutive days	lipid peroxidation increased; Fpg-specific lesions significantly increased in liver	[36]
Hepatic and renal oxidative stress enzymes	Rats, intraperitoneal 10/20/30 mg/kg	alternate days, 21 days	dose-dependent alteration; LC50 72.48 ± 10.33 mg/kg	[34]
Brain 8-OHdG, MDA, nitric oxide	Rat offspring, maternal exposure	gestation and lactation	all increased at postnatal day 60	[37]
Brain GSH, GST, GPx, SOD, CAT	Rat offspring, maternal exposure	gestation and lactation	all depleted at postnatal day 60	[37]
BALF total protein and LDH activity	Wistar rats, oropharyngeal 2/4/8 mg/kg	3 days	dose-dependent increase; consolidation at 8 mg/kg	[32]
Symptoms, body temperature, acute-phase proteins, blood neutrophils	16 healthy human volunteers	0.5, 1.0, 2.0 mg/m^3^, 4 h	concentration-dependent increase, significant from 1.0 mg/m^3^	[10]
ROS and ER stress transcripts PERK, ATF4, eIF2 alpha, CHOP	Goat mammary epithelial cells	graded dose	ROS accumulation with upregulated ER stress genes and proteins	[53]
Lipid peroxidation and chelatable iron	Mouse spermatocytes	in vivo ZnO NP exposure	both increased, driving ferroptosis	[55]

**Table 6:** Quantitative epigenetic endpoints reported after ZnO nanomaterial exposure.

Endpoint	Model	Exposure	Reported Value	References
Global 5-methylcytosine (ELISA)	MRC5 lung fibroblasts	1 µg/mL, 24 and 48 h	59% and 58.8% of control	[30]
Global 5-methylcytosine (ELISA)	MRC5 lung fibroblasts	8 µg/mL, 24 and 48 h	43% and 42% of control	[30]
DNA methyltransferase activity	MRC5 lung fibroblasts	8 µg/mL, 24 h	48% of control	[30]
Dnmt1, Dnmt3B, Dnmt3A transcripts	MRC5 lung fibroblasts	1 and 8 µg/mL, 24 h	relative expression 0.63–0.69, 0.61–0.68 and 0.98	[30]
Global 5-methylcytosine and 5-hydroxymethylcytosine	HEK-293 cells	ZnO NP exposure	5-mC reduced, 5-hmC increased	[48]
TET dioxygenase and DNMT expression	HEK-293 cells	ZnO NP exposure	TET genes significantly upregulated; DNMTs not upregulated	[48]
Global H3K9 dimethylation	HaCaT keratinocytes	20 and 50 µg/mL, 24 h	~1.5-fold higher, enriched in heterochromatin	[27]
G9a and GLP transcripts	HaCaT keratinocytes	50 µg/mL, 24 h	~50-fold and ~5-fold higher than control	[27]
Nuclear condensation and cell cycle distribution	HaCaT keratinocytes	50 µg/mL, 24 h	moderately condensed nuclei ~3.5-fold higher; G2/M 33.55% vs. 16.65%; S phase 22.33% vs. 38.83%	[27]
EZH2 protein and mRNA	T24 bladder carcinoma	5 and 10 µg/mL, 48 h	significantly decreased at both levels	[26]
Global H3K27 trimethylation and total H3	T24 bladder carcinoma	5 and 10 µg/mL, 48 h	H3K27me3 reduced; total H3 unchanged	[26]
H3K27me3 occupancy at RUNX3 promoter (ChIP-qPCR)	T24 bladder carcinoma	5 and 10 µg/mL, 48 h	markedly decreased, greatest at 10 µg/mL; RUNX3 mRNA and protein increased	[26]
Zinc chelation effect on histone axis	T24 bladder carcinoma	3 µM TPEN with ZnO NP	EZH2 and H3K27me3 increased, RUNX3 decreased	[26]
H3K9 trimethylation (CUT&Tag) with RNA-seq and ATAC-seq	Mouse preimplantation embryos and embryonic stem cells	in vivo and in vitro	H3K9me3 elevated, heterochromatin expanded, development and pluripotency genes repressed; chaetocin reversed embryotoxicity	[40]
Global DNA methylation and BDNF promoter methylation	Rat offspring brain, maternal exposure	gestation and lactation	global hypomethylation with BDNF promoter hypermethylation and reduced BDNF mRNA	[37]
Brain miR-34a and miR-29b	Rat offspring brain, maternal exposure	gestation and lactation	miR-34a increased, miR-29b decreased	[37]
Serum exosomal microRNA profile	Wistar rats, 4 mg/kg oropharyngeal	3 days	23 differentially expressed microRNAs (16 up, 7 down); 12 with more than 5-fold change	[32]
Particle-specific microRNA regulation	Chicken ovarian granulosa cells	5 µg/mL ZnO NP vs. 10 µg/mL ZnSO_4_ at equal intracellular Zn	only nanoparticle treatment regulated embryonic development-associated microRNAs	[45]
miR-342-5p and Erc1	Mouse testes and spermatocytes	in vivo exposure	miR-342-5p decreased, Erc1 derepressed, NF-kappa B activated, ferroptosis induced	[55]
S-adenosylhomocysteine (methyl donor cycle)	SMMC-7721 hepatoma, ZnO quantum dots	20 and 50 µg/mL, 24 h	13.16-fold and 23.88-fold of control	[31]

**Table 7:** Candidate biomarkers of ZnO nanoparticle exposure and effect, graded by evidence status.

Layer	Analyte	Matrix	Direction	Status	References
Mitochondrial	Membrane potential (TMRM or JC-1)	Cells	Decrease	Reproducible, dose-anchored	[3,23,24,53,54]
Mitochondrial	mtDNA copy number	Cells, tissue	Biphasic	Single model, needs replication	[3]
Mitochondrial	PGC-1 alpha, TFAM, COX IV	Cells	Decrease	Mechanistically coherent, single model	[3]
Mitochondrial	PINK1 and mitochondrial parkin	Cells	Increase	Functionally validated by knockdown	[24]
Redox	Malondialdehyde	Liver, kidney, brain	Increase	Reproducible in vivo, low specificity	[34,37,49,50]
Redox	SOD, CAT, GPx activity	Liver, kidney, brain	Decrease	Reproducible in vivo, low specificity	[34,37,49,50]
Redox	8-OHdG- and Fpg-sensitive lesions	Cells, tissue, medium	Increase	Cross-platform agreement	[31,36,37,54]
Exposure	Metallothionein, ZnT1, ZnT2	Cells, tissue	Increase	Earliest and most consistent marker	[14,15,38]
Inflammatory	Serum acute-phase proteins and neutrophils	Blood	Increase	Validated in controlled human exposure	[10,32]
Epigenetic	Global 5-methylcytosine	Cells, tissue	Decrease	Detectable at sublethal dose	[30,37,48]
Epigenetic	5-hydroxymethylcytosine and TET expression	Cells	Increase	Single model, route defining	[48]
Epigenetic	DNMT activity and Dnmt1/3B transcripts	Cells	Decrease	Single model, route defining	[30]
Epigenetic	H3K9me2 and H3K9me3	Cells, embryos	Increase	Two independent systems	[27,40]
Epigenetic	G9a and GLP transcripts	Cells	Increase	Largest reported fold change	[27]
Epigenetic	EZH2 and H3K27me3	Cells	Decrease	Zinc-dependent, chelator reversible	[26]
Epigenetic	Locus-specific promoter methylation (BDNF, HMOX1, SOD3)	Tissue, cells	Locus-dependent	Functionally linked to transcript loss	[37,48]
Noncoding RNA	Serum exosomal microRNA profile	Serum	16 up, 7 down	Minimally invasive, rodent only	[32]
Noncoding RNA	miR-5100 and coregulated isomiR cluster	Cells	Altered	Hazard ranking property demonstrated	[84]
Noncoding RNA	miR-34a, miR-29b, miR-342-5p	Brain, testes	Direction defined	Tissue-specific, mechanistically linked	[37,55]
Metabolic	S-adenosylhomocysteine	Cells	Increase	Links metabolism to methylation	[31]
Metabolic	TCA intermediates (citrate, succinate, alpha ketoglutarate)	Urine, plasma, tissue	Decrease	Reproducible across species	[33,93]

**Table 8:** Practical characteristics of the candidate biomarkers and their priority for near-term inter-laboratory validation. Cost and technical difficulty are expressed relative to the other entries in the table and reflect platform requirements rather than absolute values. Priority ratings are the assessment of the present authors and are derived from matrix accessibility, technical burden and the number of independent models in which the endpoint has been reproduced; they are not values reported in the cited sources.

Analyte	Matrix	Platform	Technical Difficulty	Relative Cost	Independent Models	Ring Trial Priority
Metallothionein, ZnT1, ZnT2 transcripts	Cells, tissue	RT-qPCR	Low	Low	Three [14,15,38]	High
Global 5-methylcytosine	Cells, tissue, blood leukocytes	Colorimetric immunoassay	Low	Low	Three [30,37,48]	High
Mitochondrial membrane potential	Cells	JC-1 or TMRM, plate reader or high content analysis	Low	Low	Five [3,23,24,53,54]	High
Acute-phase proteins, neutrophil count	Whole blood	Routine clinical chemistry and haematology	Low	Low	Controlled human exposure [10,95]	High
8-OHdG- and Fpg-sensitive lesions	Urine, serum, cells, tissue	Immunoassay, LC-MS/MS, comet assay	Moderate	Moderate	Four [31,36,37,54]	High
Malondialdehyde	Liver, kidney, brain, plasma	Thiobarbituric acid-reactive substances or HPLC	Low	Low	Four [34,37,49,50]	Medium; low specificity
SOD, CAT, GPx activity	Liver, kidney, brain, plasma	Colorimetric enzyme assay	Low	Low	Four [34,37,49,50]	Medium; low specificity
mtDNA copy number	Cells, tissue, blood	qPCR ratio to nuclear reference	Low	Low	One, biphasic [3]	Medium
DNMT activity with Dnmt1 and Dnmt3B transcripts	Cells	Colorimetric activity assay with RT-qPCR	Low	Low	One [30]	Medium
5-hydroxymethylcytosine with TET transcripts	Cells	Immunoassay with RT-qPCR	Moderate	Moderate	One [48]	Medium
Global H3K9me2, H3K9me3, H3K27me3	Cells, tissue, embryos	Immunoblot or immunoassay	Moderate	Moderate	Three [26,27,40]	Medium
G9a and GLP transcripts	Cells	RT-qPCR	Low	Low	One [27]	Medium
TCA cycle intermediates	Urine, plasma, tissue	NMR or LC-MS	Moderate	Moderate	Two species [33,93]	Medium
miR-5100 and coregulated isomiR cluster	Cells, serum	RT-qPCR after sequencing-based discovery	Moderate	Moderate	Cross material [84]	Medium
Serum exosomal microRNA profile	Serum	Exosome isolation with small-RNA sequencing	High	High	Rodent only [32]	Medium
Genome-wide DNA methylation profile	Blood leukocytes, cells	Methylation array	High	High	Feasible in exposed workers [97,98]	Medium
PGC-1 alpha, TFAM, COX IV protein	Cells, tissue	Immunoblot	Moderate	Moderate	One [3]	Low
PINK1 and mitochondrial parkin	Cells	Subcellular fractionation with immunoblot	High	Moderate	One, knockdown validated [24]	Low
Locus-specific H3K27me3 occupancy	Cells	ChIP-qPCR	High	High	One [26]	Low
S-adenosylhomocysteine	Cells, plasma	LC-MS/MS	Moderate	Moderate	One [31]	Low
Transcriptome and microRNA profile	Cells, tissue, blood	RNA sequencing	High	High	Multiple [14,38,39]	Low

## Data Availability

No new data were created or analyzed in this study. Data sharing is not applicable to this article.
